# Improving Adolescent Health and Nutrition in Sub‐Saharan Africa: The Time to Act Is Now

**DOI:** 10.1111/mcn.70038

**Published:** 2025-04-22

**Authors:** Cristina Álvarez Sánchez, Vilma Tyler

**Affiliations:** ^1^ UNICEF Child Nutrition and Development Section, Headquarters New York New York USA

Adolescence, particularly the early adolescent years (ages 10–14), is a time of extraordinary physical, cognitive and socio‐emotional growth and development (Patton et al. 2016; National Academies of Sciences 2019). It offers a unique opportunity to promote good nutrition and health, offset early childhood nutritional deprivations (Norris et al. 2022), and establish lifelong healthy lifestyle habits (Neufeld et al. 2022). Good nutrition during adolescence results in better educational outcomes and lifelong benefits (Bundy et al. 2017).

Yet, an estimated 250 million adolescents live in countries facing a triple burden of infectious diseases, non‐communicable diseases, and injuries (Patton et al. 2016), and one in seven adolescents experience mental health disorders (WHO 2024).

Young people in Sub‐Saharan Africa are disproportionally affected—due to poverty, limited educational opportunities, and social instability—with mortality rates twice as high as in other regions (Ross et al. 2021). Considering that Sub‐Saharan Africa is home to over 250 million adolescents, over 20% of the world's total, and that this proportion is set to increase to 24% by 2030 (Population Reference Bureau PRB 2021; United Nations, Department of Economic and Social Affairs, Population Division 2019), it is critical to act now to improve adolescent health and nutrition in the region.

Schools represent the best platform through which to deliver nutrition interventions to prevent all forms of malnutrition in adolescents, given their high reach and regular contact with students throughout many years. The school system is a particularly relevant setting, given that enrolment rates have increased worldwide, including in Sub‐Saharan Africa.

Global reports on adolescent health and nutrition have highlighted significant gaps in data, research, investment, and adolescent‐friendly interventions (Patton et al. 2016; Patton et al. 2022). This is particularly true for younger adolescents in Sub‐Saharan Africa. Existing surveys—including the Demographic and Health Surveys, Multiple Indicator Cluster Surveys, and the Global School‐Based Student Health Survey—largely exclude 10–14‐year‐old adolescents and fail to assess the school environment and policies that influence their health and well‐being.

To address knowledge gaps, the African Research, Implementation Science, and Education (ARISE) Network, supported by UNICEF, have conducted novel school‐based studies of young adolescents’ (of 10–15 years) health and nutrition across five urban cities in Burkina Faso, Ethiopia, South Africa, Sudan, and Tanzania (Shinde, Noor, et al. 2023). This study, together with a global systematic review of school‐based health and nutrition interventions, is presented in the *Maternal and Child Nutrition* Special Issue on Improving Nutrition, Health, and Well‐being of Young Adolescents in the African Context (Berhane et al. 2023; Costa et al. 2024; Drysdale et al. 2023; Madzorera et al. 2023; Millogo et al. 2024; Noor et al. 2024; Partap et al. 2023; Sando et al. 2024; Shinde, Noor, et al. 2023; Shinde, Perumal, et al. 2023; Shinde, Wang, et al. 2023; Wang et al. 2023).

This Special Issue also offers comprehensive tools for analyzing the school food and nutrition environment, which can be used or adapted to support the development of strategies and prioritization of efforts to prevent and address multiple forms of malnutrition among school‐aged adolescents. The articles also outline methods for conducting a comprehensive analysis of adolescents’ nutritional status, identifying its determinants, assessing school food and nutrition environments, and reviewing existing policies, laws, strategies, and programs within the country context. Using this analysis, national and subnational governments can design or refine policies and strategies to address childhood malnutrition, while also identifying gaps in data and evidence.

Authors of this Special Issue found that young adolescents in the five Sub‐Saharan Africa cities suffer from multiple forms of malnutrition and socioemotional challenges. Anaemia affects a large proportion of young adolescents in Sudan (25.0%), Burkina Faso (36.2%), and Tanzania (58.3%) (Partap et al. 2023); Millogo et al. 2024). In Ethiopia, 8% of young adolescents were found to have overweight (Drysdale et al. 2023); while it is a relatively low prevalence, it is indicative of the region's emerging triple burden of malnutrition. Mental health issues such as anxiety and depression were prevalent in one in eight adolescents in Burkina Faso, Ethiopia, and Tanzania (Shinde, Perumal, et al. 2023).

Poor diets and food insecurity underlie many of these issues. The diets of young adolescents in Sub‐Saharan Africa are notably low in nutrient‐dense foods—such as fruits, vegetables, and animal‐source foods—and high in refined grains (Madzorera et al. 2023; Berhane et al. 2023). Adolescents frequently consume unhealthy food products—such as sweets, sugar‐sweetened beverages (SSBs) and unhealthy snacks—, oftentimes buying them from school vendors (Noor et al. 2024; Berhane et al. 2023). They are also largely inactive, engaging in physical activity only about 1.5 days a week.

Their nutritional status was also found to have an important influence on the time when girls get their first menstrual period—known as menarche. Underweight and stunted girls were less likely to experience menarche, while overweight girls were more likely to experience earlier menarche (Costa et al. 2024). Both delayed and accelerated puberty carry health and developmental risks. However, an early menarche, raises serious concerns due to its association with early sexual initiation, increased risk of sexually transmitted diseases, early pregnancy, and subsequent adverse health and developmental outcomes, in a region that already has record adolescent pregnancies.

The Special Issue's authors also evaluated school health and nutrition programming, services and policies across the five countries, finding significant gaps. Less than half of the schools assessed had a health and nutrition curriculum, and many schools lacked essential health, nutrition, and WASH services (Noor et al. 2024). Many countries in Sub‐Saharan Africa have policies that outline the basic elements of school health and nutrition programmes. Oftentimes however, their implementation is hampered by insufficient funding, lack of implementation and monitoring frameworks, lack of intersectoral coordination, and lack of capacities within schools, as seen in Tanzania (Sando et al. 2024).

School assessments revealed a high prevalence of food vendors selling unhealthy products, such as deep‐fried foods and SSBs, around schools (Berhane et al. 2023; Noor et al. 2024). In Ethiopia, students expressed how they would like to buy more of the ‘cheap and sweet foods’ sold by vendors, but they could only afford small amounts (Berhane et al. 2023). With rising populations and incomes, the lack of regulation of children's food environments is likely to lead to an increase in consumption of unhealthy products and a higher number of overweight adolescents in the region. Regulation of food environments in and around schools is urgently needed to prevent this growing public health issue.

The Special Issue also presents a systematic review of the impact of school‐based health and nutrition interventions to address the double burden of malnutrition in low‐ and middle‐income countries (Shinde, Wang, et al. 2023). The review found mixed results. Multi‐component interventions and nutrition education showed positive impacts on nutritional or diet‐related outcomes. However, many interventions were not optimally designed: two‐thirds had no theoretical framing, three‐fourths were short in duration, and most did not engage students and parents in their design and implementation.

Adolescents in Sub‐Saharan African are the key to the region's development, but they suffer from multiple forms of malnutrition and mental health challenges. School health and nutrition interventions hold promise to improving adolescent health and wellbeing, but significant gaps in implementation remain across the five studies countries. By prioritizing adequate funding, capacity development, and actively involving adolescents and their communities, policymakers, researchers, and programmers can address the challenges highlighted in this Special Issue. The time to act is now. Ensuring the health, nutrition, and well‐being of young adolescents in Sub‐Saharan Africa will not only ensure they grow and develop to their full potential but also drive transformative change for the region, creating healthier, more prosperous generations.

## Author Contributions

Cristina Álvarez Sánchez drafted the initial manuscript. Vilma Tyler provided critical revisions. All authors approved the final version.

## Conflicts of Interest

The authors declare no conflicts of interest.

## Data Availability

The authors have nothing to report.
